# *Lentinula edodes*-Derived Polysaccharide Alters the Spatial Structure of Gut Microbiota in Mice

**DOI:** 10.1371/journal.pone.0115037

**Published:** 2015-01-21

**Authors:** Xiaofei Xu, Xuewu Zhang

**Affiliations:** College of Light Industry and Food Sciences, South China University of Technology, Guangzhou, China; Massachusetts General Hospital, UNITED STATES

## Abstract

*Lentinula edodes*-derived polysaccharides possess many therapeutic characteristics, including anti-tumor and immuno-modulation. The gut microbes play a critical role in modulation of immune function. However, the impact of *Lentinula edodes*-derived polysaccharides on the gut microbes have not yet been explored. In this study, high-throughput pyrosequencing technique was employed to investigate the effects of a new heteropolysaccharide L2 from *Lentinula edodes* on microbiota diversity and composition of small intestine, cecum, colon and distal end of colon (feces) in mice. The results demonstrated that along mouse intestine the microbiota exhibit distinctly different space distribution. L2 treatment reduced the diversity and evenness of gut microbiota along the intestine, especially in the cecum and colon. In the fecal microbial communities, the decrease of Bacteroidetes by significantly increasing Proteobacteria were observed, which were characterized by the increased *Helicobacteraceae* and reduced S24-7 at family level. Some OTUs, corresponding to *Bacteroides acidifaciens*, *Alistipes* and *Helicobacter suncus*, were found to be significantly increased in L2 treated-mice. In particular, 4 phyla Chloroflexi, Gemmatimonadetes, Nitrospirae and Planctomycetes are exclusively present in L2-treated mice. This is helpful for further demonstrating healthy action mechanism of *Lentinula edodes*-derived polysaccharide L2.

## Introduction

*Lentinula edodes* is the second most popular edible mushroom in the world. With the enormous development in the field of purification and structure determination, many types of polysaccharides have been obtained from the fruit body of *Lentinula edodes* [1]. Most of the Lentinula edodes-derived polysaccharides have been shown to possess many therapeutic applications, such as cancer, depressed immune function, and hyperlipidemia [2]. However, these polysaccharides, known as nondigestible carbohydrates, are not fully digested in the upper gut. It has become clear that the healthy benefits of nondigestible carbohydrates are attributed to the contribution of gut microbiota, i.e. fermenting nondigestible carbohydrates to produce gut-absorbable metabolites and to stimulate proliferation of certain bacteria [3,4]. Recent evidence has shown that several types of non-digestible carbohydrates have a major influence on microbial community composition both in the short and long term [5]. For example, Martinez et al.[6] demonstrated that resistant starches types 2 and 4 exhibit functional differences in their effect on human fecal microbiota composition. Marín-Manzano et al. [7] assessed the modulatory influence of novel galacto-oligosaccharides derived from lactulose (GOS-Lu) in rat gut microbiota.

On the other hand, dysbiosis of gut microbiota is closely related to human health and diseases [8]. Numerous researches have showed that the gut microbes play a critical role in the development of immune system and modulation of immune function [9,10]. So, it is important to understand the community profiles and system characteristics of gut microbiota after administration of non-digestible polysaccharides. As the first medicinal mushroom in clinical field, the immune-modulating and anti-tumor characteristics of polysaccharides derived from *Lentinula edodes* have been intensively investigated [1]. However, it is less clear about how *Lentinula edodes*-derived polysaccharides impact the characteristics and distribution of gut microbiota. In our previous study [11], a new heteropolysaccharide L2 with immunostimulating activity was isolated from the fruit body of *Lentinula edodes*. Chemical characterization indicated that L2 consists of glucose (87.5%), galactose (9.6%), and arabinose (2.8%), with a molecular weight of 26 KDa. The primary linkages are the 1→ or 1→6 glycosidic linkages accounted for 44.5% and 1→2 or 1→4 glycosidic linkages accounted for 17.9%. Other linkages include (1→3)-linked, (1→2, 3)-linked, (1→2, 4)-linked, (1→3, 4)-linked, (1→3, 6)-linked, and (1→2, 3, 4)-linked through glucose, which account for about 37.6% of all linkages in the molecule. Especially, L2 does not possess a triple-helical conformation by Congo red assay [11]. The goal of this study is to investigate the effects of L2 on microbiota diversity and composition along the mouse intestine using a high-throughput pyrosequencing technique. It is expected to provide foundation for *Lentinula edodes*-derived polysaccharide L2 in understanding healthy action mechanism and discovering potential side effects.

## Materials and Methods

### Reagents and experimental design

*Lentinula edodes*-derived polysaccharide L2 was prepared as described before [8]. Specific pathogen-free male C57BL/6 mice (8-week old) were obtained from Vital River Laboratory Animal Technology Co. Ltd. (Beijing, China). All chemical reagents were at analytical grade.

The mice were kept at a temperature of 22°C and 12-h light/dark cycles environment for at least two weeks before use, and fed on the same batch of standard laboratory diet to minimize the variation of environment factors. The experiments were approved by the Animal Care Welfare Committee of Guangzhou University of Chinese Medicine. Adequate measures were taken to minimize pain of experimental animals. Mice were divided into two groups for 28 consecutive days: (1) *Lentinula edodes*-derived polysaccharide L2 treatment groups (gavage administration of L2 40mg/kg body weight, n = 7) (Na groups), which were reared in the same cage; (2) normal groups (gavage administration with the same volume of sterile physiological saline, 8-week old, n = 7) (N groups), which were reared in the same cage.

Fresh fecal samples were collected on the last day and immediately frozen in liquid nitrogen before storage at −80°C for further analysis. Then, mice were weighted and peripheral blood samples were taken and stored at 4°C. Subsequently, mice were sacrificed by cervical dislocation, and spleen and thymus were immediately removed under sterile environment and weighted. The small intestine, cecum and colon contents were sterilely collected separately and immediately stored at −80°C till for further analysis.

### DNA Extraction and Pyrosequencing

Six fecal samples and 3 intestinal content samples were randomly selected from each group for 16S rRNA gene pyrosequencing. Genomic DNA was extracted from small intestine, cecum and colon contents as well as fecal samples by using the Soil DNA kit (Omega Bio-Tek, Inc., GA, USA) according to the manufacturer’s instructions.

Pyrosequencing was carried out according to previously described [12–13]. PCR amplification of the V1-V3 region of bacterial 16 S rRNA gene was performed using universal primers (533R 5′-TTACCGCGGCTGCTGGCAC-3′, 27F 5′-AGAGTTTGATCCTGGCTCAG-3′) incorporating the FLX Titanium adapters and a sample barcode sequence. The forward primer (B-27F) was 5′-*TATCCCCTGTGTGCCTTGGCAGT CGACT*AGAGTTTGATCCTGGCTCAG-3′, where the sequence of the B adaptor is shown in italics and underlined. The reverse primer (A-533R) was 5′-*ATCTCATCCCTGCGTGTCTC CGACGACT*NNNNNNNNTTACCGCGGCTGCTGGCAC-3′, where the sequence of the A adaptor is shown in italics and underlined and the Ns represent an eight-base sample specific barcode sequence. Briefly, Each 20 μL PCR reaction included 4 μL of 5 *FastPfu Buffer, 2 μL of 2.5 mM dNTPs, 0.4 μL of Forward Primer (5 mM), 0.4 μL of Reverse Primer (5 mM),0.5 μL of DNA template, 0.4 μL of Fastfu Polymerase, and added ddH2O to make up the final volume to 20 μL. The cycling parameters were as follows: 95°C for 2 min; 25 cycles of 95°C for30 s, 56°C for 30 s, 72°C for 30 s with a final extension at 72°C for 5 min. Duplicate PCR products were pooled. Then they were visualized on agarose gels (2% in TBE buffer) containing ethidium bromide, and purified using the AXYGEN gel extraction kit (Axygen, USA). Amplicon DNA concentrations were measured using the Quant-iT PicoGreen dsDNA reagent and kit (Invitrogen, Germany) and was quality controlled on an Agilent 2100 bioanalyzer (Agilent, USA). Following quantitation, the amplicon from each reaction mixture were pooled in equimolar ratios based on concentration and subjected to emulsion PCR to generate amplicon libraries, as recommended by 454 Life Sciences. Pyrosequencing was performed by a 454 Life Sciences Genome Sequencer FLX Titanium instrument (Roche) (Shanghai Majorbio, Shanghai, China).

All pyrosequencing reads were filtered according to barcode and primer sequences. The resulting sequences were further screened and filtered for quality and length. Sequences that were less than 200 bp, contained ambiguous characters, contained over two mismatches to the primers, had an average quality score below 25 or contained mononucleotide repeats of over 8 bp were removed using Mothur software package (version 1.28.0) (command trim.seqs)(http://www.mothur.org/wiki/Main_Page) [14,15]. A total of 69,700 high quality sequences were obtained after the filtering process.

### Bioinformatic Analysis

The high-quality sequences were assigned to samples according to barcodes. Sequences were aligned in accordance with SILVA alignment (Bacterial SILVA database, SILVA version 111, http://www.arb-silva.de/) using kmer searching (http://www.mothur.org/wiki/Align.seqs) in Mothur (command align.seqs)[16]. The aligned sequences were further trimmed and the redundant reads were eliminated using UCHIME for further denoising and removal of potentially chimeric sequences [17]. Unique sequences were clustered into operational taxonomic units (OTUs) using a 97% identity cut-off and the furthest neighbor clustering algorithm in Mothur (commands: dist.seqs; cluster; classfy.seqs), which were assigned to taxonomy in accordance with SILVA 111 at 80% confidence level using Naïve Bayesian classifier [15,16,18]. OTUs were used for alpha diversity (Shannon,Simpson, and evenness (J indices)), richness (Chao1 and Ace) using Mothur. Community structure comparisons with principal coordinate analysis (PCA) based on weighted Unifac distance, Jaccard tree clustering analysis, and heatmap were performed using Mothur and R software package (http://www.R-project.org).

### Statistical Analysis

The Mann-Whitney test and student’s-test were performed using SPSS19.0. P-values < 0.05 were considered significant unless otherwise stated.

## Results

### Diversity of the bacterial communities along the gastrointestinal tract in normal and L2-treated mice

Multiplex pyrosequencing of covering the V1-V3 hypervariable regions of 16S rRNA gene was employed to characterize the bacterial community diversity along the mice gastrointestinal tract. Following all denoising and filtering steps, a dataset consisting of 117497 (mean±S.D., 7833±1682) reads from N groups and 106268 (7084±2056) reads from Na groups were used in the final analysis (Table 1). Based on a sequence similarity of greater than 97%, an average of 1862 and 1611 OTUs were defined for N and Na groups, respectively (Table 1).

**Table 1:** 16S rRNA gene sequencing statistics from normal and L2-treated C57BL/6 mice.

Sample ID*	**Reads**	**OTUs**	**Chao1**	**Ace**	**Coverage**
N1	6785	1687	4277	7309	0.823876
N2	9879	2145	5039	7777	0.867902
N4	6574	1858	4659	7829	0.814573
N5	8494	2218	5351	9217	0.83141
N6	9019	1757	4344	6630	0.880585
N7	6443	2051	5099	8532	0.788297
Na1	6439	2352	7326	13286	0.735673
Na2	5726	1547	4589	8637	0.806322
Na3	5860	1831	5267	10311	0.777133
Na4	6290	1535	4005	6474	0.836089
Na6	6212	2009	5396	9665	0.770444
Na7	6951	2068	5927	11358	0.793411
N21	8851	1372	2519	3608	0.917523
N41	11130	485	893	1232	0.976999
N61	10120	888	1575	2566	0.952273
Na41	12305	1058	1789	2457	0.957009
Na61	10030	973	1792	2548	0.95005
Na71	7177	656	1250	1756	0.950258
N22	5766	1916	4491	7176	0.78616
N42	6582	2399	5873	10110	0.756001
N62	6019	2166	4940	8231	0.764413
Na42	5547	1708	4949	8267	0.785109
Na62	5908	1602	4592	8314	0.807549
Na72	5791	1817	5436	11148	0.775168
N23	6188	1933	4625	7860	0.795895
N43	7715	2368	5899	9538	0.802074
N63	7932	2698	6343	11151	0.776727
Na43	7295	1460	4270	7674	0.863605
Na63	9866	2023	5261	9469	0.860328
Na73	4871	1536	4611	8768	0.771094

* N×1 (Na×1), N×2 (Na×2) and N×3 (Na×3) correspond to small intestine, cecum and colon samples in mice Nx (Nax), respectively.

**Figure 1:**
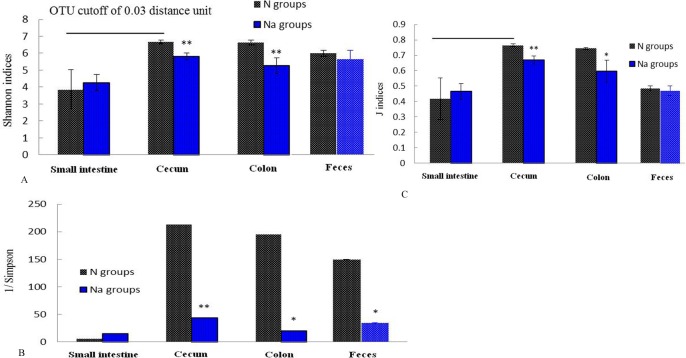
Alpha diversity of fecal microbiota in normal (N) and L2-treated mice (Na).*, p<0.05;**p<0.01. The index of evenness (J indices) was calculated with the formula E = H/ln(S), where H is the Shannon index and S is the number of OTU in that animal.

Table 2 showed that cecum and colon displayed higher diversity of microbiota than that of small intestine in N groups (p<0.01). For the estimated richness (Chao1 and Ace) of fecal, small intestine, cecum or colon microbiota, no significant differences were observed in N and Na groups. But L2 treatment significantly decreased the amount of OTUs in cecum or colon microbiota (p<0.05), compared with N groups. Fig. 1 demonstrated that the shannon and evenness indices are similar in N and Na groups for small intestine or fecal microbiota. However, L2 treatment significantly increased the simpson index in cecum, colon or fecal microbiota, compared with N groups (p<0.05 or p<0.01), i.e. higher degree of convergence in Na groups than in N groups.

**Table 2:** Diversity of gut microbiota in normal mice (N) and L2-treated mice (Na).

**Sample type**	**groups**	**OTUs**	**Chao1**	**Ace**
		**Mean±SD**	**Mean±SD**	**Mean±SD**
feces	N	1652.7±216.7	4795.8±436.2	7882.3±907.1
	Na	1890.3±318.3	5418.3±1149.5	9955.2±2329.3
small intestine	N	915 ± 362.6	1662.3 ± 666.7	2468.7 ± 972.4
	Na	895.7 ± 173.0	1610.3 ± 254.8	2253.7 ± 353.9
cecum	N	2160.3 ± 197.2**	5101.3 ± 575.6**	8505.7 ± 1213.4**
	Na	1709 ± 87.8*	4992.3 ± 345.9	9243 ± 1347.2
colon	N	2333 ± 313.3**	5622.3 ± 728.1**	9516.3 ± 1343.6**
	Na	1673 ± 249.4*	4714 ± 411.1	8637 ± 738.6

* P<0.05

**, p<0.01, student t-test

**Figure 2:**
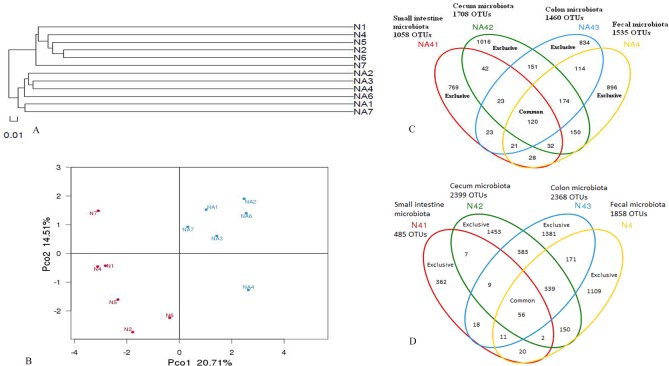
Clustering (A) and weighted unifrac PCA (B) analysis of fecal microbiota, as well as OTUs overlaps of a L2-treated mice Na4 (C) and a normal mice N4 (D) along the intestinal tract.

For fecal microbiota, clustering analysis displayed the formation of two major clusters corresponding to normal and L2-treated groups, respectively (Fig. 2A). Weighted Unifrac analysis revealed that each mouse was obviously different from all others and a high degree of variation between individuals existed (Fig. 2B). The first principal coordinate (PC1), which accounted for 20.71% of variance in the data, can completely separate N from L2-treated (Na) groups (Fig. 2B). The second principal coordinate (PC2, 14.51% of variance in the data) can separate N from L2-treated (Na) groups with >80% of accuracy, except for the mice N7 in normal groups and Na4 in L2-treated groups (Fig. 2B). By random selection of an individual mouse in each group (Na4 and N4), the diversity of bacterial populations along the intestinal tract was compared. The amount of OTUs existed exclusively in small intestine, cecum, colon and fecal microbiota were 769, 1016, 834,896 for Na4, and 362, 1453, 1381, 1109 for N4, respectively; whereas the amount of their common OTUs were 120 and 56, respectively (Fig. 2C; Fig. 2D).

### Spatial structures of the bacterial communities from small intestine, cecum and colon contents in normal and L2-treated mice

**Figure 3:**
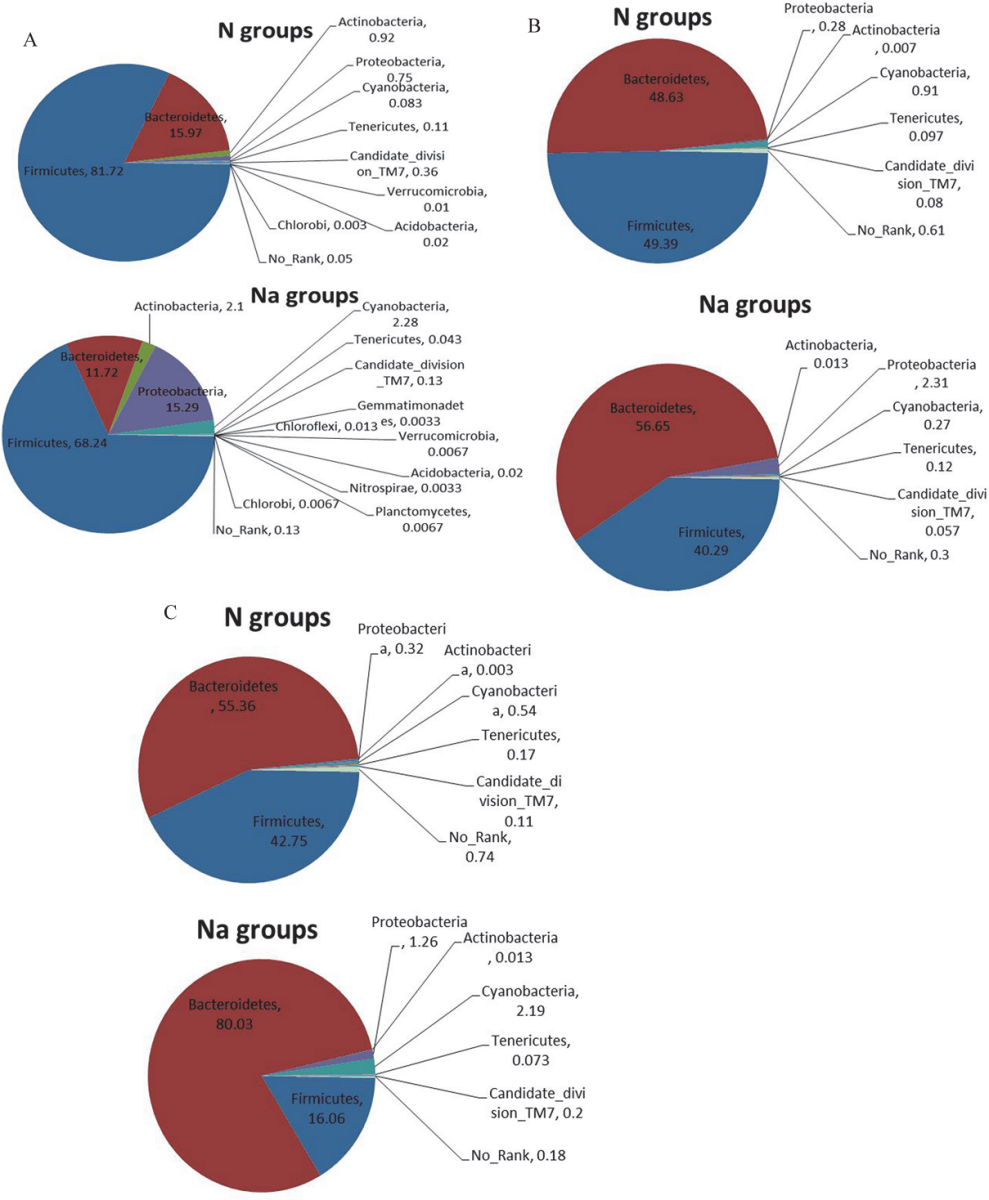
Comparison of small intestine (A), cecum (B) and colon (C) microbiota at phylum level.

For small intestine microbiota (Fig. 3A), the prevalent bacterial communities (%) in N groups are Firmicutes (F) and Bacteroidetes (B), in total >97 and F/B = 5.12; while the top bacterial communities (%) in L2-treated groups include 4 phyla: Firmicutes, Bacteroidetes, Proteobacteria and Actinobacteria, in total >97% and F/B = 5.82. That is, L2 treatment reduced the abundance of Firmicutes and Bacteroidetes with similar ratio (F/B), but significantly increased the proportions of Proteobacteria (15.29% vs 0.75%, p<0.05) and Actinobacteria (2.10% vs. 0.92%, p<0.05), compared with N groups. In particular, 4 phyla Chloroflexi, Gemmatimonadetes, Nitrospirae and Planctomycetes are exclusively present in L2-treated mice, not detected in normal mice.

For cecum microbiota (Fig. 3B), the prevalent bacterial communities are also 2 phyla (in total >96%): Firmicutes (F) and Bacteroidetes (B), with F/B = 1.02 and F/B = 0.71 in N and L2-treated groups, respectively. Specifically, L2 induced the decrease of Firmicutes and the increase of Bacteroidetes. Especially, L2 remarkably increased the proportion of Proteobacteria (2.31% vs. 0.28%, P = 0.08), and significantly decreased the abundance of Cyanobacteria (0.27% vs. 0.91%, P<0.05), compared with normal mice. In cecum microbial cummunities, the dominant bacteria groups were Bacteroidaceae, Rikenellaceae, Ruminococcaceae, Lachnospiraceae, and S24-7. Microbial communities from 6 mice were clustered into 2 groups: Na42, Na62; N22, N42, N62, Na72, according to the similarity in relative abundance at family level (Figure A in S1 File).

Similar to cecum microbiota, the most abundant bacterial communities in colon microbiota (Fig. 3C) remains 2 phyla (in total >96%): Firmicutes (F) and Bacteroidetes (B), but the ratio F/B is greatly changed as 0.77 and 0.20 in N and L2-treated groups, respectively. In other words, L2 significantly elevated the proportions of Bacteroidetes (80.03% vs. 55.36%, p<0.05), and reduced the level of Firmicutes (16.06% vs. 42.75%, p<0.05), compared with N groups. Moreover, more Proteobacteria were observed in L2-treated groups than in N groups (1.26% vs. 0.32%, p<0.05). However, the dominant bacterial populations were consisted of S24-7, Lachnospiraceae, Rikenellaceae, Bacteroidaceae, Ruminococcaceae, and Prevotellaceae. Microbial communities from 6 mice were clustered into 2 groups: Na43, Na63; N43, N63, N23, Na73, which could be further clustered into 3 subgroups: Na43, Na63; N43, N63; N23, Na73, according to the similarity in relative abundance at family level (Figure B in S1 File).

### L2 treatment shifts the composition of fecal microbiota

**Figure 4:**
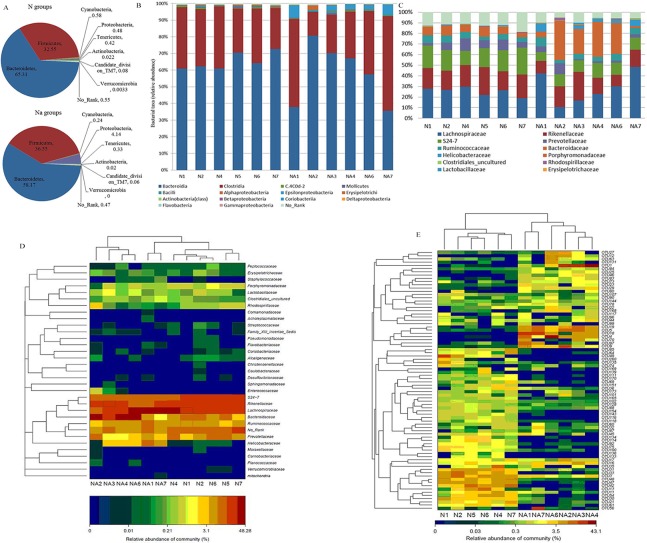
Composition of fecal microbiota at phylum (A), class (B), family (C, D), and OTUs (E) levels.

Next, we focus on the impacts of L2 treatment on fecal microbiota in mice. Phylum-level comparison of fecal microbiota in normal and L2-treated mice showed that Firmicutes (F) and Bacteroidetes (B) are prevalent communities, in total 97.8%, F/B = 0.5, in N groups; while in L2-treated groups the prevalent communities include 3 phyla: Firmicutes, Bacteroidetes and Proteobacteria, in total 98.86%, F/B = 0.63 (Fig. 4A). It is noted that Proteobacteria is very significantly increased (4.14% vs. 0.043%, p<0.01), and Cyanobacteria is decreased (0.24% vs. 0.58%, p = 0.055) in L2-treated groups, compared with N groups (Table 3).

**Table 3:** Phylum-level comparison of fecal microbiota in normal (N) and L2-treated mice (Na).

**Phylum**	**N groups (n = 6) mean±SD%**	**Na groups (n = 6) mean±SD%**	**P values**
Actinobacteria	0.022±0.026	0.020±0.020	0.93
Bacteroidetes	65.31±5.12	58.17±18.17	0.52
Candidate division TM7	0.082±0.082	0.060±0.068	0.63
Cyanobacteria	0.58±0.22	0.24±0.25	0.055
Firmicutes	32.55±5.49	36.55±16.60	0.75
Proteobacteria	0.478±0.21	4.14±2.03	0.004
Tenericutes	0.42±0.25	0.35±0.33	0.52
Verrucomicrobia	0.0033±0.0052	0	0.14

Mann Whitney test.

At class level (Fig. 4B), the bacterial communities in N groups are consisted of two major classes: Bacteroidia (B) (65.2%) and Clostridia (C) (32%), B+C = 97.2% and B/C = 2; while the bacterial populations in L2-treated mice primarily include 3 phyla: Bacteroidia (58.1%), Clostridia (35.5%) and Epsilonproteobacteria (3.6%), in total 97.2%, B+C = 93.6% and B/C = 1.6. The major differences compared with normal groups are the significant increases of Epsilonproteobacteria (p<0.05) and moderate increase of Bacili (0.35% vs 0.75%, p = 0.1).

The composition of fecal microbiota at family level in N and L2-treated groups and the heatmap analysis of their relative abundance are displayed in Fig. 4C and Fig. 4D, respectively. In N groups, the most abundant families are composed of 6 populations: Lachnospiraceae, Rikenellaceae, S24-7, Prevotellaceae, Ruminococcaceae and Bacteroidaceae, totally >80%. In L2-treated groups, the top populations adds up to 7 families: Lachnospiraceae, Rikenellaceae, S24-7, Prevotellaceae, Ruminococcaceae, Bacteroidaceae and Helicobacteraceae, totally >90%. The major variations are: Helicobacteraceae (3.6% vs 0.02%, p<0.05) are significantly increased, while S24-7 is significantly reduced (11.2% vs 19.5, p<0.01) in L2-treated groups, compared with N groups (Table 4). Strikingly, the species *Helicobacter suncus* was significantly increased from 0 to 2.34% after L2 treatment.

**Table 4:** Family-level comparison of fecal microbiota in normal (N) and L2-treated mice (Na).

**Bacteria family**	**N groups (n = 6) mean±SD %**	**Na groups (n = 6) mean ± SD %**
Lachnospiraceae	25.24 ± 4.05	28.50 ± 14.70
Rikenellaceae	20.68 ± 3.12	16.70 ± 5.92
S24-7	19.48 ± 3.92	11.20 ± 1.76**
Prevotellaceae	7.37 ± 3.24	3.85 ± 3.49
Ruminococcaceae	5.59 ± 1.37	5.61 ± 2.01
Bacteroidaceae	6.70 ± 1.89	21.40 ± 14.80
Helicobacteraceae	0.018 ± 0.012	3.65 ± 2.58*
Porphyromonadaceae	0.71 ± 0.32	0.41 ± 0.24
Clostridiales uncultured	0.22 ± 0.11	0.35 ± 0.26
Rhodospirillaceae	0.38 ± 0.19	0.45 ± 0.89
Lactobacillaceae	0.30 ± 0.20	0.53 ± 0.39
Erysipelotrichaceae	0.13 ± 0.12	0.14 ± 0.08

Mann Whitney test.

*, P<0.05

**, P<0.01.

Furthermore, top 80 OTUs (Table A in S1 File) of relative abundance presented in N and L2-treated groups were selected for comparison. Fig. 4E demonstrated that the top 80 OTUs exhibited two clusters and can separate fecal microbiota of both groups, consistent with clustering analysis above. In particular, the microbial communities of Na4, Na6, Na7, N2, N4, N6 were also clustered into 2 subgroups: Na4, Na6; N2, N4, N6, Na7, which were consistent with the results in cecum and colon (Figure A and Figure B in S1 File). Compared with N groups, L2 can significantly (p<0.05) facilitate the growth of 15 OTUs (Table B in S1 File), such as OTU1 (0.004% vs 7.71%), OTU4 (0.004% vs 4.09%), OTU5 (0.024% vs 2.54%), OTU18 (0% vs 2.31%), OTU19 (0.024% vs 2.70%). Among them, OTU1 and OTU4 are attibuted to species of *Bacteroides acidifaciens*, OTU5 and OTU19 belong to species of *Alistipes uncultured Bacteroidaceae bacterium*, while OTU18 is the species *Helicobacter suncus*. On the other hand, compared with N groups, 34 OTUs are significantly (p<0.05) repressed in L2-treated groups (Table B in S1 File). The representative OTUs include: OTU3 (2.1% vs 0.58%), OTU11 (1.1% vs 0.133%), OTU13 (1.52% vs 0.014%), OTU30 (1.19% vs 0.024%), OTU31 (1.68% vs 0.17%), OTU33 (1.85% vs 0.008%), OTU42 (2.16% vs 0.003%), OTU47 (2.18% vs 0.017%), OTU48 (2.29% vs 0.027%), OTU49(1.31% vs 0%), OTU61 (0.98% vs 0.023%), OTU100 (0.522% vs 0.033%). Except for OTU49, OTU133, OTU139 belonging to Lachnospiraceae (Phylum of Firmicutes), other OTUs belong to the order Bacteroidales, and the majority of them are from the genus *Alistipes* (especially in the species of *Alistipes uncultured Bacteroidaceae bacterium*) and the family S24-7 (especially in the species of *S24-7 mouse gut metagenome*).

## Discussion

Gut microbiota has been recognized as being implicated in human health and disease, but feces do not fully reflect microbial ecology in the intestine [19]. It is essential to investigate the characteristics and distribution of the microbial community along the mouse gastrointestinal tract [20]. Non-digestible carbohydrates have great impact on the gut microbiota [4,5]. More and more evidences indicated that non-digestible carbohydrates affect the profiles of intestinal microbial community depending on the structure of polysaccharides such as glycosidic linkage and monosaccharide composition [4–5,21–23]. Mushroom polysaccharides have been proposed as new prebiotic resource and have attractive attentions from researchers [3,24]. *Letinula edodes*-derived polysaccharides have been intensively investigated for their potentially therapeutic applications [1, 25]. However, the influence of *Letinula edodes*-derived polysaccharides on gut microbiota was rarely reported. The present study, explored the impact of a mushroom polysaccharide, *Lentinula edodes*-derived polysaccharide L2, on microbiota diversity and composition along the mouse intestine using a high-throughput pyrosequencing technique.

Overall, as described above, along the small intestine, cecum, colon and distal colon (feces), the microbiota composition changes from Firmicutes (F)-dominant to Bacteroidetes (B)-dominant structure in normal and L2-treated mice. The most significantly difference happens in the colon, the ratio F/B varies from 0.77 to 0.2. This suggests the massive presence of responsive bacteria to polysaccharide L2 in colon, and these responders primarily belong to Bacteroidetes population, which is consistent with previous findings in African children consuming the high-plant polysaccharide diets [26]. It is well known that plant polysaccharides are not digested by human enzymes, but are processed to absorbable short chain fatty acids (SCFA) by gut bacteria. Bacteroidetes can use a series of membrane protein complexes, termed Sus-like systems, to metabolize many complex carbohydrates [27]. For example, B. thetaiotaomicron and B. ovatus were found to have capability of utilizing nearly all of the major plant and host glycans [28]. Interestingly, a previous study revealed that non-digestible polysaccharide (oat β-glucan) was not degraded while passing the small intestine in human [29], suggesting L2 could reach colon where L2 was fermented by gut microbiota. The phylum Bacteroidetes were enriched for carbohydrate metabolic pathways, whereas the phylum Firrmicutes possessed a disproportionately fewer number of polysaccharide-degrading enzymes [30,31]. So, the enrichment of Bacteroidetes in the colon in L2-treated mice could be related to degradation of L2 in mouse intestine.

Another common feature is that Proteobacteria is significantly increased in all microbiota from small intestine, cecum, colon and distal colon (feces), up to 20.4, 8.3, 3.9 and 8.6 folds, respectively, speculating that this might be associated with the immuno-stimulating activity of polysaccharide L2. For example, Proteobacteria can induce specific IgA response to regulate the maturation of intestinal microbiota [32]. *Bacteroides acidifaciens* was found to promote IgA production in the large intestine by inducing germinal center formation and increasing the number of IgA+ B cells [33]. However, in contrast to the previous results that plant-derived non-digestible carbohydrates increased the diversity of fecal microbiota [26, 34–35], L2 reduced the richness, diversity, and evenness of microbial communities in cecum and colon. The possible reasons could be due to the direct stimulation of L2 on intestinal epithelial cells (IECs) that can secret cytokines and regulate the host immune responses to the gut microbiota upon activated by fungal polysaccharides [36–38], which finally shaped the communities of gut microbiota [39]. The supernatant of illeostomic content from ileostomic patients taking oat β-glucan showed immuno-stimulating effects both in small intestinal (INT407) and colon (HT29) cell lines [29], indicating that the TLR2-involved L2 might have immuno-stimulating effects on enterocytes because enterocytes expressed toll-like receptors [11,40]. In SPF mice, the clusters of low richness and diversity in fecal microbiota characterized by the decreased Firmicutes and the increased Bacteroidetes, Proteobacteria were observed recently, which were found to be associated with low grade intestinal inflammation [41]. Similarly, Bacteroidaceae, Lachnospiraceae, Rikenellaceae, Prevotellaceae as well as Ruminococcaceae were correlated to low grade intestinal inflammation [41]. L2 could activate pro-inflammatory cytokines secretion in immune cells in vitro [11]. In intestine, two important components of the intestinal immune system are the IECs that form a physical barrier and the gut-associated lymphoid tissue (GALT) system consisting of various immune cells (T-cells, B-cells, and intestinal macrophages)[42]. After oral administration, IECs might transduced signals from L2 to adjacent immune cells of the intestinal immune system via pattern recognition receptors (PRRs), like toll-like receptors (TLRs), and induced the transcription of pro-inflammatory cytokines. Taken together, the shifts of gut microbiota in L2-treated mice might be attributed in part to the immuno-stimulating activity of L2. The results emphasized the need for further research to explain effects of L2 on IECs and gut bacteria groups.

In particular, by comparing top 80 OTUs presented in N and L2-treated groups, several species or OTUs, including species of *Bacteroides acidifaciens* and genus of *Alistipes*, were found to be significantly increased after L2 treatment. The more abundant *Bacteroides acidifaciens* and *Alistipes* might be linked to L2 degradation in mouse intestine. Such as, *Alistipes putredinis* can degrade fiber and glucosinolates [43]. *Alistipes finegoldii* were suggested to be involved in the metabolism of glycans [44]. Different resources of non-digestible polysaccharides have different chemical structure, which might influenced the profiles of populations and metabolites of bacteria groups. Hull-less barley cultivars, barley cultivars with hulls, oat cultivars, oat groats differed in β-glucan, non-starch polysaccharide, and resistant starch have been used to explore their effects on microbial communities and SCFA profiles in vitro and in vivo. The results revealed a complex interactions between different polysaccharides and the intestinal bacteria, indicating that the glycosidic linkage type might influence the intestinal microbial communities and metabolites [21–23,45–49]. The characteristics that some intestinal microbial groups might prefer to utilize specific polysaccharides and produce different metabolites profiles are of fundamental importance. Understanding the mechanism would provide opportunity for designing non-digestible polysaccharides formula to manipulate the intestinal microbial communities and their metabolisms.

Fungal polysaccharides, especially mushroom polysaccharides, mostly have anti-inflammatory activity [50]. Mizuno et al demonstrated that Lentinan, *Lentinula edodes*-derived β-1→3,1→6-glucan, showed anti-inflammatory activity on an in vitro gut inflammation model[37]. Furthermore, Lentinan significantly ameliorated DSS-induced colitis, indicating the potential application in treatment of gut inflammatory diseases such as ulcerative colitis and Crohn’s disease [51]. Inflammatory bowel disease (IBD) is characterized by the dysbiosis of gut microbiota [52]. L2 showed great influences on the gut microbiota and immune stimulating activity. Further studies are urgently needed to elucidate the possibility in therapeutic application of L2 to IBD [53–54].

In conclusion, the spatial distribution of gut microbiota from mouse small intestine, cecum, colon and distal colon (feces) is remarkably different. The overall composition shifts from Firmicutes (F)-dominant to Bacteroidetes (B)-dominant structure, with F/B values as 5.12, 1, 0.77, 0.50 in normal mice and 5.82, 0.71, 0.20, 0.63 in L2-treated mice. L2 treatment decreased the diversity and evenness of gut microbiota along the intestine, especially in the cecum and colon. Other significantly changed populations in response to L2 treatment include: Proteobacteria, *Bacteroides acidifaciens, Alistipes* and *Helicobacter suncus*. In particular, 4 phyla Chloroflexi, Gemmatimonadetes, Nitrospirae and Planctomycetes are exclusively present in L2-treated mice. It warrants further study on the association of these structural features with healthy benefits of polysaccharide L2, such as the treatment of dysbiosis-related diseases, IBD.

## Supporting Information

S1 File Table A. The taxonomical information of the top 80 OTUs in fecal microbiota. Table B. Alterations in the top 80 OTUs responding to L2 treatment. Figure A. Comparison of cecum microbial communities at family level. Figure B. Comparison of colon microbial communities at family level.
(DOC)
