# Supplementary material for: Lentinula edodes-Derived Polysaccharide Alters the Spatial Structure of Gut Microbiota in Mice
Source: PLoS One. 2015 Jan 21;10(1):e0115037. doi: 10.1371/journal.pone.0115037 (PMC4301806; doi:10.1371/journal.pone.0115037)
Supplement: S1 File — Table B. Alterations in the top 80 OTUs responding to L2 treatment. Figure A. Comparison of cecum microbial communities at family level. Figure B. Comparison of colon microbial communities at family level. (DOC) [file pone.0115037.s001.doc]

**Supporting Information**

Table A The taxonomical information of the top 80 OTUs in fecal microbiota

| OTU name | phylum | class | order | family | genus | species |
| --- | --- | --- | --- | --- | --- | --- |
| OTU1 | Bacteroidetes | Bacteroidia | Bacteroidales | Bacteroidaceae | *Bacteroides* | *Bacteroides_acidifaciens* |
| OTU3 | Bacteroidetes | Bacteroidia | Bacteroidales | Rikenellaceae | *Alistipes* |  |
| OTU4 | Bacteroidetes | Bacteroidia | Bacteroidales | Bacteroidaceae | *Bacteroides* | *Bacteroides_acidifaciens* |
| OTU5 | Bacteroidetes | Bacteroidia | Bacteroidales | Rikenellaceae | *Alistipes* | *Alistipes_uncultured_Bacteroidaceae_bacterium* |
| OTU9 | Bacteroidetes | Bacteroidia | Bacteroidales | Prevotellaceae |  |  |
| OTU11 | Bacteroidetes | Bacteroidia | Bacteroidales | Prevotellaceae | *Prevotella* |  |
| OTU12 | Bacteroidetes | Bacteroidia | Bacteroidales | Bacteroidaceae | *Bacteroides* | *Bacteroides_sp._S-18* |
| OTU13 | Firmicutes | Clostridia | Clostridiales | Lachnospiraceae |  |  |
| OTU16 | Bacteroidetes | Bacteroidia | Bacteroidales | Rikenellaceae | *Alistipes* | *Alistipes_uncultured_Bacteroidaceae_bacterium* |
| OTU17 | Bacteroidetes | Bacteroidia | Bacteroidales |  |  | *S24-7_mouse_gut_metagenome* |
| OTU18 | Proteobacteria | Epsilonproteobacteria | Campylobacterales | Helicobacteraceae | *Helicobacter* | *Helicobacter_suncus* |
| OTU19 | Bacteroidetes | Bacteroidia | Bacteroidales | Rikenellaceae | *Alistipes* | *Alistipes_uncultured_Bacteroidaceae_bacterium* |
| OTU21 | Bacteroidetes | Bacteroidia | Bacteroidales |  |  | *S24-7_mouse_gut_metagenome* |
| OTU22 | Bacteroidetes | Bacteroidia | Bacteroidales | Rikenellaceae | *Alistipes* |  |
| OTU23 | Bacteroidetes | Bacteroidia | Bacteroidales |  |  |  |
| OTU25 | Bacteroidetes | Bacteroidia | Bacteroidales |  |  | *S24-7_mouse_gut_metagenome* |
| OTU27 | Bacteroidetes | Bacteroidia | Bacteroidales | Bacteroidaceae | *Bacteroides* | *Bacteroides_sp._S-18* |
| OTU29 | Bacteroidetes | Bacteroidia | Bacteroidales |  |  | *S24-7_mouse_gut_metagenome* |
| OTU30 | Bacteroidetes | Bacteroidia | Bacteroidales |  |  |  |
| OTU31 | Bacteroidetes | Bacteroidia | Bacteroidales | Prevotellaceae |  |  |
| OTU32 | Bacteroidetes | Bacteroidia | Bacteroidales |  |  | *S24-7_mouse_gut_metagenome* |
| OTU33 | Bacteroidetes | Bacteroidia | Bacteroidales | Bacteroidaceae | *Bacteroides* | *Bacteroides_acidifaciens* |
| OTU35 | Firmicutes | Clostridia | Clostridiales | Lachnospiraceae |  |  |
| OTU36 | Bacteroidetes | Bacteroidia | Bacteroidales | Rikenellaceae | *Alistipes* |  |
| OTU42 | Bacteroidetes | Bacteroidia | Bacteroidales | Rikenellaceae | *Alistipes* | *Alistipes_uncultured_Bacteroidaceae_bacterium* |
| OTU44 | Bacteroidetes | Bacteroidia | Bacteroidales |  |  | *S24-7_mouse_gut_metagenome* |
| OTU45 | Bacteroidetes | Bacteroidia | Bacteroidales |  |  |  |
| OTU46 | Bacteroidetes | Bacteroidia | Bacteroidales | Rikenellaceae | *Alistipes* | *Alistipes_uncultured_Bacteroidaceae_bacterium* |
| OTU47 | Bacteroidetes | Bacteroidia | Bacteroidales | Rikenellaceae | *Alistipes* | *Alistipes_uncultured_Bacteroidaceae_bacterium* |
| OTU48 | Bacteroidetes | Bacteroidia | Bacteroidales | Rikenellaceae | *Alistipes* | *Alistipes_uncultured_Bacteroidaceae_bacterium* |
| OTU49 | Firmicutes | Clostridia | Clostridiales | Lachnospiraceae |  |  |
| OTU54 | Bacteroidetes | Bacteroidia | Bacteroidales | Prevotellaceae | *Prevotella* |  |
| OTU56 | Firmicutes | Clostridia | Clostridiales | Lachnospiraceae |  |  |
| OTU60 | Bacteroidetes | Bacteroidia | Bacteroidales |  |  | *S24-7_mouse_gut_metagenome* |
| OTU61 | Bacteroidetes | Bacteroidia | Bacteroidales |  |  |  |
| OTU63 | Firmicutes | Clostridia | Clostridiales | Lachnospiraceae |  | *Lachnospiraceae_Clostridium_sp._Clone-27* |
| OTU64 | Bacteroidetes | Bacteroidia | Bacteroidales |  |  | *S24-7_mouse_gut_metagenome* |
| OTU69 | Bacteroidetes | Bacteroidia | Bacteroidales |  |  | *S24-7_mouse_gut_metagenome* |
| OTU70 | Bacteroidetes | Bacteroidia | Bacteroidales | Rikenellaceae |  |  |
| OTU73 | Bacteroidetes | Bacteroidia | Bacteroidales |  |  | *S24-7_mouse_gut_metagenome* |
| OTU74 | Firmicutes | Clostridia | Clostridiales | Lachnospiraceae |  | *Lachnospiraceae_Clostridium_sp._Clone-27* |
| OTU75 | Bacteroidetes | Bacteroidia | Bacteroidales | Bacteroidaceae | *Bacteroides* | *Bacteroides_acidifaciens* |
| OTU78 | Firmicutes | Clostridia | Clostridiales | Lachnospiraceae |  |  |
| OTU80 | Firmicutes | Clostridia | Clostridiales | Lachnospiraceae |  |  |
| OTU83 | Bacteroidetes | Bacteroidia | Bacteroidales |  |  | *S24-7_mouse_gut_metagenome* |
| OTU84 | Bacteroidetes | Bacteroidia | Bacteroidales |  |  | *S24-7_mouse_gut_metagenome* |
| OTU85 | Bacteroidetes | Bacteroidia | Bacteroidales |  |  |  |
| OTU87 | Firmicutes | Clostridia | Clostridiales | Lachnospiraceae |  |  |
| OTU88 | Bacteroidetes | Bacteroidia | Bacteroidales | Rikenellaceae | *Alistipes* |  |
| OTU90 | Firmicutes | Clostridia | Clostridiales | Ruminococcaceae |  |  |
| OTU92 | Bacteroidetes | Bacteroidia | Bacteroidales |  |  | *S24-7_mouse_gut_metagenome* |
| OTU96 | Firmicutes | Clostridia | Clostridiales | Lachnospiraceae |  | *Lachnospiraceae_Clostridium_sp._Clone-27* |
| OTU97 | Firmicutes | Clostridia | Clostridiales | Lachnospiraceae |  |  |
| OTU99 | Firmicutes | Clostridia | Clostridiales | Lachnospiraceae |  |  |
| OTU100 | Bacteroidetes | Bacteroidia | Bacteroidales |  |  | *S24-7_mouse_gut_metagenome* |
| OTU101 | Proteobacteria | Alphaproteobacteria | Rhodospirillales | Rhodospirillaceae | *Thalassospira* | *Thalassospira_uncultured_rumen_bacterium* |
| OTU102 | Bacteroidetes | Bacteroidia | Bacteroidales |  |  | *S24-7_mouse_gut_metagenome* |
| OTU107 | Bacteroidetes | Bacteroidia | Bacteroidales |  |  | *S24-7_mouse_gut_metagenome* |
| OTU111 | Bacteroidetes | Bacteroidia | Bacteroidales |  |  | *S24-7_mouse_gut_metagenome* |
| OTU114 | Bacteroidetes | Bacteroidia | Bacteroidales | Bacteroidaceae | *Bacteroides* | *Bacteroides_acidifaciens* |
| OTU116 | Bacteroidetes | Bacteroidia | Bacteroidales |  |  |  |
| OTU117 | Bacteroidetes | Bacteroidia | Bacteroidales |  |  | *S24-7_mouse_gut_metagenome* |
| OTU118 | Bacteroidetes | Bacteroidia | Bacteroidales | Porphyromonadaceae |  |  |
| OTU128 | Bacteroidetes | Bacteroidia | Bacteroidales |  |  | *S24-7_mouse_gut_metagenome* |
| OTU131 | Firmicutes | Clostridia | Clostridiales | Lachnospiraceae |  |  |
| OTU133 | Firmicutes | Clostridia | Clostridiales | Lachnospiraceae |  |  |
| OTU134 | Bacteroidetes | Bacteroidia | Bacteroidales | Rikenellaceae | *Alistipes* |  |
| OTU135 | Bacteroidetes | Bacteroidia | Bacteroidales | Rikenellaceae | *Alistipes* | *Alistipes_uncultured_Bacteroidaceae_bacterium* |
| OTU136 | Firmicutes | Clostridia | Clostridiales | Lachnospiraceae |  |  |
| OTU139 | Firmicutes | Clostridia | Clostridiales | Lachnospiraceae |  |  |
| OTU143 | Bacteroidetes | Bacteroidia | Bacteroidales |  |  |  |
| OTU144 | Bacteroidetes | Bacteroidia | Bacteroidales |  |  | *S24-7_mouse_gut_metagenome* |
| OTU151 | Bacteroidetes | Bacteroidia | Bacteroidales | Prevotellaceae | *Prevotella* |  |
| OTU154 | Bacteroidetes | Bacteroidia | Bacteroidales |  |  |  |
| OTU165 | Bacteroidetes | Bacteroidia | Bacteroidales |  |  | *S24-7_mouse_gut_metagenome* |
| OTU168 | Bacteroidetes | Bacteroidia | Bacteroidales | Rikenellaceae |  |  |
| OTU169 | Firmicutes | Clostridia | Clostridiales | Lachnospiraceae |  |  |
| OTU170 | Bacteroidetes | Bacteroidia | Bacteroidales |  |  |  |
| OTU173 | Bacteroidetes | Bacteroidia | Bacteroidales | Bacteroidaceae | *Bacteroides* |  |
| OTU260 | Firmicutes | Clostridia | Clostridiales | Ruminococcaceae |  |  |

Table B Alterations in the top 80 OTUs responding to L2 treatment

| OTU name | N1,% | N2,% | N4,% | N5,% | N6,% | N7,% | Na1,% | Na2,% | Na3,% | Na4,% | Na6,% | Na7,% | p value |
| --- | --- | --- | --- | --- | --- | --- | --- | --- | --- | --- | --- | --- | --- |
| OTU1 | 0.000 | 0.000 | 0.000 | 0.012 | 0.011 | 0.000 | 0.264 | 13.133 | 11.331 | 20.572 | 0.950 | 0.014 | 0.003 |
| OTU3 | 2.786 | 0.028 | 2.175 | 1.789 | 3.648 | 2.157 | 0.714 | 0.175 | 0.717 | 1.256 | 0.580 | 0.008 | 0.037 |
| OTU4 | 0.000 | 0.000 | 0.000 | 0.012 | 0.011 | 0.000 | 2.718 | 3.755 | 3.089 | 2.846 | 11.961 | 0.172 | 0.003 |
| OTU5 | 0.029 | 0.000 | 0.000 | 0.047 | 0.033 | 0.031 | 1.739 | 4.715 | 4.693 | 2.719 | 1.352 | 0.019 | 0.025 |
| OTU9 | 0.029 | 0.000 | 0.015 | 0.012 | 0.033 | 0.031 | 2.190 | 7.579 | 0.392 | 0.986 | 1.143 | 0.016 | 0.016 |
| OTU11 | 0.265 | 0.003 | 2.282 | 1.377 | 1.774 | 0.900 | 0.419 | 0.000 | 0.154 | 0.064 | 0.161 | 0.002 | 0.037 |
| OTU12 | 0.000 | 0.000 | 0.000 | 0.000 | 0.000 | 0.000 | 0.140 | 3.580 | 0.751 | 0.175 | 2.849 | 0.041 | 0.002 |
| OTU13 | 0.059 | 0.001 | 1.050 | 2.213 | 5.755 | 0.016 | 0.016 | 0.017 | 0.051 | 0.000 | 0.000 | 0.000 | 0.043 |
| OTU16 | 0.486 | 0.005 | 0.654 | 1.554 | 0.843 | 0.512 | 0.248 | 1.345 | 2.338 | 1.701 | 0.821 | 0.012 | 0.423 |
| OTU17 | 0.796 | 0.008 | 0.243 | 0.600 | 0.887 | 1.055 | 0.326 | 2.620 | 0.290 | 0.366 | 0.113 | 0.002 | 0.423 |
| OTU18 | 0.000 | 0.000 | 0.000 | 0.000 | 0.000 | 0.000 | 6.383 | 0.017 | 2.662 | 2.512 | 2.238 | 0.032 | 0,002 |
| OTU19 | 0.015 | 0.000 | 0.030 | 0.071 | 0.000 | 0.031 | 1.444 | 2.323 | 4.932 | 2.432 | 1.272 | 0.018 | 0.016 |
| OTU21 | 0.280 | 0.003 | 0.319 | 0.871 | 1.319 | 1.676 | 0.047 | 0.035 | 0.119 | 0.079 | 0.000 | 0.000 | 0.025 |
| OTU22 | 0.000 | 0.000 | 0.502 | 0.118 | 0.133 | 0.372 | 0.047 | 0.384 | 0.000 | 0.286 | 0.918 | 0.013 | 0.747 |
| OTU23 | 0.029 | 0.000 | 0.030 | 0.035 | 0.100 | 0.016 | 0.683 | 0.000 | 0.563 | 0.541 | 0.274 | 0.004 | 0.229 |
| OTU25 | 0.000 | 0.000 | 0.030 | 0.000 | 0.000 | 0.016 | 0.171 | 0.664 | 0.341 | 1.002 | 0.209 | 0.003 | 0.009 |
| OTU27 | 0.118 | 0.001 | 0.015 | 0.035 | 0.122 | 0.031 | 0.047 | 2.235 | 0.887 | 0.000 | 1.932 | 0.028 | 0.337 |
| OTU29 | 0.015 | 0.000 | 0.000 | 0.000 | 0.011 | 0.000 | 0.404 | 0.332 | 0.410 | 1.431 | 0.837 | 0.012 | 0.006 |
| OTU30 | 2.314 | 0.023 | 1.552 | 1.095 | 1.220 | 0.916 | 0.109 | 0.017 | 0.017 | 0.000 | 0.000 | 0.000 | 0.006 |
| OTU31 | 1.430 | 0.014 | 1.810 | 2.343 | 3.005 | 1.459 | 0.311 | 0.489 | 0.017 | 0.095 | 0.113 | 0.002 | 0.037 |
| OTU32 | 0.103 | 0.001 | 0.365 | 0.047 | 0.011 | 0.295 | 0.047 | 0.017 | 0.085 | 0.270 | 0.080 | 0.001 | 0.630 |
| OTU33 | 3.183 | 0.032 | 2.069 | 2.849 | 1.630 | 1.304 | 0.000 | 0.017 | 0.000 | 0.032 | 0.000 | 0.000 | 0.004 |
| OTU35 | 0.000 | 0.000 | 3.803 | 0.624 | 0.299 | 0.047 | 0.171 | 0.000 | 0.751 | 0.000 | 0.000 | 0.000 | 0.305 |
| OTU36 | 0.000 | 0.000 | 1.186 | 0.341 | 0.499 | 0.916 | 0.295 | 0.087 | 0.017 | 0.064 | 0.193 | 0.003 | 0.336 |
| OTU42 | 1.002 | 0.010 | 2.632 | 4.003 | 2.240 | 3.042 | 0.000 | 0.000 | 0.017 | 0.000 | 0.000 | 0.000 | 0.005 |
| OTU44 | 0.472 | 0.005 | 0.441 | 0.306 | 0.000 | 0.186 | 0.326 | 0.035 | 0.648 | 1.526 | 0.032 | 0.000 | 0.688 |
| OTU45 | 0.324 | 0.003 | 0.350 | 0.283 | 0.798 | 0.388 | 0.808 | 0.017 | 0.341 | 0.000 | 0.000 | 0.000 | 0.197 |
| OTU46 | 0.000 | 0.000 | 0.000 | 0.000 | 0.000 | 0.016 | 0.248 | 0.943 | 0.973 | 0.413 | 0.064 | 0.001 | 0.005 |
| OTU47 | 3.331 | 0.034 | 2.297 | 3.096 | 1.597 | 2.732 | 0.031 | 0.017 | 0.051 | 0.000 | 0.000 | 0.000 | 0.006 |
| OTU48 | 3.272 | 0.033 | 2.540 | 3.002 | 1.996 | 2.871 | 0.047 | 0.035 | 0.034 | 0.016 | 0.032 | 0.000 | 0.016 |
| OTU49 | 6.352 | 0.064 | 0.335 | 0.494 | 0.610 | 0.000 | 0.000 | 0.000 | 0.000 | 0.000 | 0.000 | 0.000 | 0.007 |
| OTU54 | 0.133 | 0.001 | 2.921 | 1.330 | 2.351 | 1.009 | 0.047 | 0.000 | 0.017 | 0.000 | 0.000 | 0.000 | 0.009 |
| OTU56 | 0.044 | 0.000 | 0.000 | 0.012 | 0.067 | 0.031 | 0.000 | 0.052 | 0.000 | 0.016 | 0.048 | 0.001 | 0.871 |
| OTU60 | 0.029 | 0.000 | 0.350 | 0.177 | 0.233 | 0.248 | 0.000 | 0.000 | 0.085 | 0.079 | 0.080 | 0.001 | 0.107 |
| OTU61 | 0.766 | 0.008 | 0.685 | 1.719 | 1.375 | 1.335 | 0.000 | 0.052 | 0.085 | 0.000 | 0.000 | 0.000 | 0.009 |
| OTU63 | 0.354 | 0.004 | 0.076 | 0.141 | 0.022 | 0.000 | 0.311 | 0.489 | 0.546 | 0.525 | 2.930 | 0.042 | 0.025 |
| OTU64 | 0.486 | 0.005 | 0.061 | 0.353 | 0.920 | 2.701 | 0.016 | 0.000 | 0.017 | 0.016 | 0.000 | 0.000 | 0.015 |
| OTU69 | 0.103 | 0.001 | 0.198 | 0.047 | 0.344 | 1.071 | 0.124 | 0.000 | 0.034 | 0.000 | 0.032 | 0.000 | 0.036 |
| OTU70 | 0.000 | 0.000 | 0.000 | 0.000 | 0.000 | 0.000 | 1.755 | 0.000 | 2.986 | 0.556 | 0.016 | 0.000 | 0.022 |
| OTU73 | 0.560 | 0.006 | 0.076 | 0.000 | 0.233 | 0.000 | 0.202 | 0.262 | 0.563 | 0.731 | 0.386 | 0.006 | 0.092 |
| OTU74 | 1.120 | 0.011 | 0.000 | 0.130 | 0.011 | 0.000 | 0.016 | 0.000 | 0.290 | 0.079 | 0.225 | 0.003 | 0.628 |
| OTU75 | 0.973 | 0.010 | 0.639 | 0.765 | 0.499 | 0.372 | 0.000 | 0.105 | 0.000 | 0.000 | 0.000 | 0.000 | 0.005 |
| OTU78 | 0.162 | 0.002 | 0.122 | 0.094 | 0.333 | 0.171 | 0.373 | 0.210 | 0.222 | 0.397 | 0.515 | 0.007 | 0.078 |
| OTU80 | 0.000 | 0.000 | 0.000 | 0.012 | 0.000 | 0.000 | 0.419 | 0.035 | 0.000 | 0.668 | 0.080 | 0.001 | 0.021 |
| OTU83 | 0.059 | 0.001 | 0.000 | 0.012 | 0.033 | 0.016 | 0.186 | 0.279 | 0.751 | 0.397 | 0.580 | 0.008 | 0.025 |
| OTU84 | 0.029 | 0.000 | 0.061 | 0.059 | 0.067 | 0.062 | 0.124 | 0.314 | 0.461 | 0.270 | 0.016 | 0.000 | 0.229 |
| OTU85 | 0.427 | 0.004 | 0.350 | 0.341 | 0.432 | 2.639 | 0.016 | 0.000 | 0.000 | 0.000 | 0.000 | 0.000 | 0.005 |
| OTU87 | 0.000 | 0.000 | 0.137 | 0.059 | 0.377 | 0.217 | 0.031 | 0.070 | 0.017 | 0.016 | 0.097 | 0.001 | 0.521 |
| OTU88 | 0.221 | 0.002 | 0.213 | 0.506 | 0.333 | 0.264 | 0.093 | 0.087 | 0.119 | 0.270 | 0.064 | 0.001 | 0.109 |
| OTU90 | 0.221 | 0.002 | 0.167 | 0.024 | 0.022 | 0.047 | 0.202 | 0.052 | 0.119 | 0.556 | 0.499 | 0.007 | 0.200 |
| OTU92 | 0.796 | 0.008 | 0.578 | 0.777 | 0.421 | 0.217 | 0.016 | 0.052 | 0.034 | 0.032 | 0.032 | 0.000 | 0.037 |
| OTU96 | 0.427 | 0.004 | 0.198 | 0.035 | 0.000 | 0.000 | 0.450 | 0.035 | 0.512 | 0.413 | 0.177 | 0.003 | 0.172 |
| OTU97 | 0.000 | 0.000 | 0.030 | 0.071 | 0.266 | 0.000 | 3.044 | 0.052 | 0.188 | 0.000 | 0.000 | 0.000 | 0.798 |
| OTU99 | 0.796 | 0.008 | 0.426 | 0.106 | 0.000 | 0.000 | 0.186 | 0.472 | 0.000 | 0.000 | 0.000 | 0.000 | 0.393 |
| OTU100 | 1.017 | 0.010 | 0.441 | 0.730 | 0.765 | 0.171 | 0.062 | 0.017 | 0.068 | 0.000 | 0.048 | 0.001 | 0.025 |
| OTU101 | 0.280 | 0.003 | 0.015 | 0.377 | 0.310 | 0.310 | 0.062 | 1.886 | 0.119 | 0.032 | 0.097 | 0.001 | 0.521 |
| OTU102 | 0.265 | 0.003 | 0.183 | 0.224 | 0.177 | 0.341 | 0.062 | 0.087 | 0.068 | 0.064 | 0.097 | 0.001 | 0.037 |
| OTU107 | 0.310 | 0.003 | 0.106 | 0.259 | 0.133 | 0.062 | 0.062 | 0.035 | 0.102 | 0.191 | 0.338 | 0.005 | 0.688 |
| OTU111 | 0.221 | 0.002 | 0.228 | 0.130 | 0.355 | 0.745 | 0.000 | 0.000 | 0.017 | 0.000 | 0.000 | 0.000 | 0.005 |
| OTU114 | 0.236 | 0.002 | 0.654 | 1.189 | 1.109 | 0.233 | 0.000 | 0.017 | 0.000 | 0.000 | 0.000 | 0.000 | 0.005 |
| OTU116 | 0.295 | 0.003 | 0.274 | 0.389 | 0.565 | 0.202 | 0.000 | 0.000 | 0.000 | 0.000 | 0.000 | 0.000 | 0.002 |
| OTU117 | 0.604 | 0.006 | 0.639 | 0.000 | 0.033 | 0.031 | 0.590 | 0.000 | 0.171 | 0.302 | 0.000 | 0.000 | 0.463 |
| OTU118 | 0.265 | 0.003 | 0.335 | 0.459 | 0.820 | 0.233 | 0.016 | 0.017 | 0.017 | 0.000 | 0.032 | 0.000 | 0.024 |
| OTU128 | 0.310 | 0.003 | 0.228 | 0.506 | 0.421 | 0.140 | 0.031 | 0.017 | 0.102 | 0.079 | 0.048 | 0.001 | 0.037 |
| OTU131 | 0.029 | 0.000 | 0.000 | 0.000 | 0.067 | 0.000 | 0.047 | 0.244 | 0.051 | 0.000 | 1.980 | 0.028 | 0.097 |
| OTU133 | 0.000 | 0.000 | 0.669 | 0.565 | 0.155 | 0.000 | 0.000 | 0.000 | 0.000 | 0.000 | 0.000 | 0.000 | 0.059 |
| OTU134 | 0.015 | 0.000 | 0.091 | 0.977 | 1.120 | 0.124 | 0.000 | 0.000 | 0.000 | 0.000 | 0.016 | 0.000 | 0.021 |
| OTU135 | 2.388 | 0.024 | 0.076 | 0.247 | 0.067 | 0.186 | 0.047 | 0.017 | 0.068 | 0.048 | 0.000 | 0.000 | 0.025 |
| OTU136 | 0.000 | 0.000 | 0.867 | 0.824 | 0.067 | 0.000 | 0.000 | 0.000 | 0.000 | 0.000 | 0.000 | 0.000 | 0.059 |
| OTU139 | 0.059 | 0.001 | 0.015 | 0.059 | 1.663 | 0.000 | 0.000 | 0.000 | 0.000 | 0.000 | 0.000 | 0.000 | 0.007 |
| OTU143 | 0.339 | 0.003 | 0.563 | 0.271 | 0.266 | 0.233 | 0.000 | 0.000 | 0.000 | 0.000 | 0.000 | 0.000 | 0.002 |
| OTU144 | 0.236 | 0.002 | 0.030 | 0.130 | 0.166 | 0.062 | 0.078 | 0.140 | 0.188 | 0.175 | 0.290 | 0.004 | 0.337 |
| OTU151 | 0.029 | 0.000 | 0.974 | 0.330 | 0.432 | 0.295 | 0.000 | 0.000 | 0.000 | 0.000 | 0.000 | 0.000 | 0.007 |
| OTU154 | 0.383 | 0.004 | 0.046 | 0.459 | 0.266 | 0.310 | 0.031 | 0.000 | 0.000 | 0.000 | 0.000 | 0.000 | 0.005 |
| OTU165 | 0.236 | 0.002 | 0.304 | 0.212 | 0.444 | 0.372 | 0.000 | 0.070 | 0.017 | 0.048 | 0.000 | 0.000 | 0.016 |
| OTU168 | 0.000 | 0.000 | 0.000 | 0.094 | 0.067 | 0.000 | 0.016 | 1.834 | 0.154 | 0.382 | 0.982 | 0.014 | 0.022 |
| OTU169 | 0.000 | 0.000 | 0.000 | 0.047 | 1.197 | 0.466 | 0.016 | 0.000 | 0.034 | 0.000 | 0.000 | 0.000 | 0.284 |
| OTU170 | 0.251 | 0.003 | 0.715 | 0.165 | 0.200 | 0.729 | 0.000 | 0.000 | 0.000 | 0.000 | 0.000 | 0.000 | 0.002 |
| OTU173 | 0.206 | 0.002 | 0.152 | 0.047 | 0.599 | 0.310 | 0.000 | 0.454 | 0.119 | 0.000 | 0.386 | 0.006 | 0.423 |
| OTU260 | 1.857 | 0.019 | 0.000 | 0.012 | 0.000 | 0.435 | 0.000 | 0.000 | 0.000 | 0.000 | 0.000 | 0.000 | 0.022 |


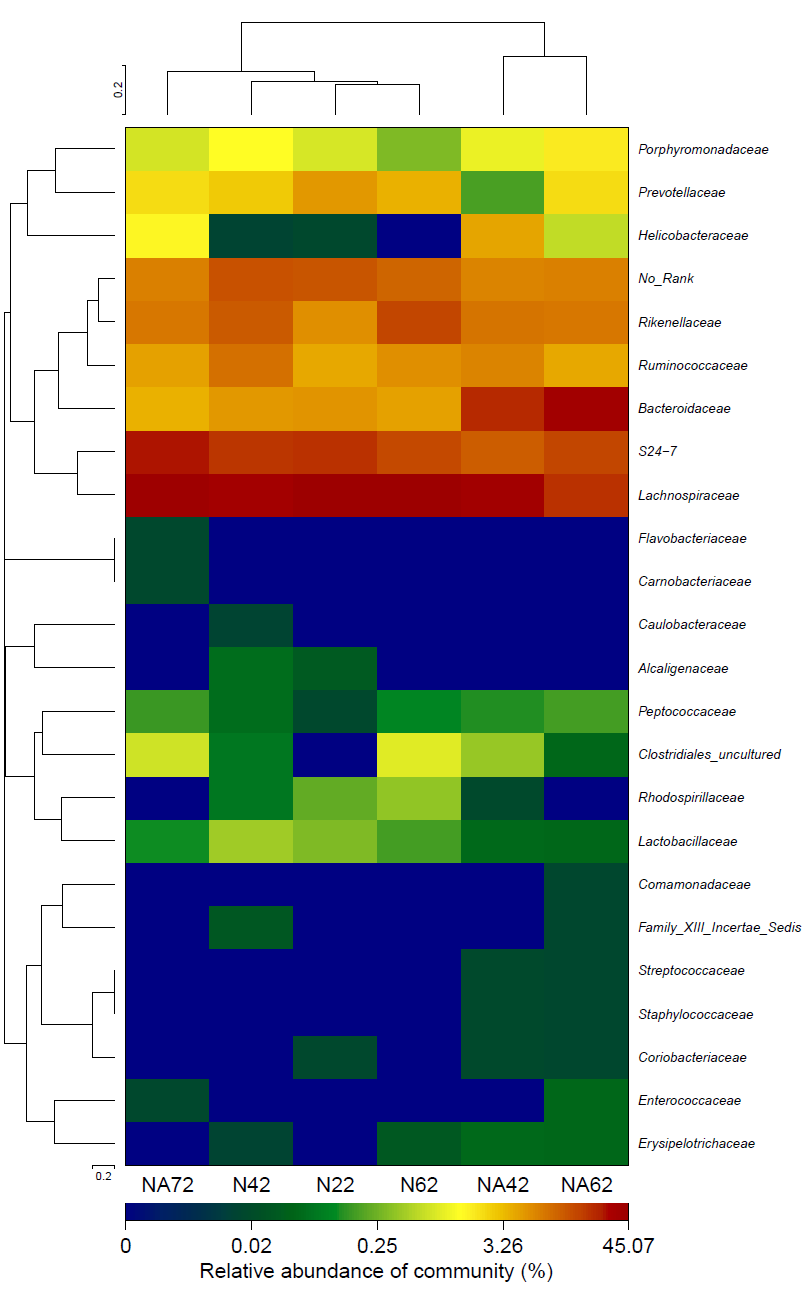


Figure A Comparison of cecum microbial communities at family level


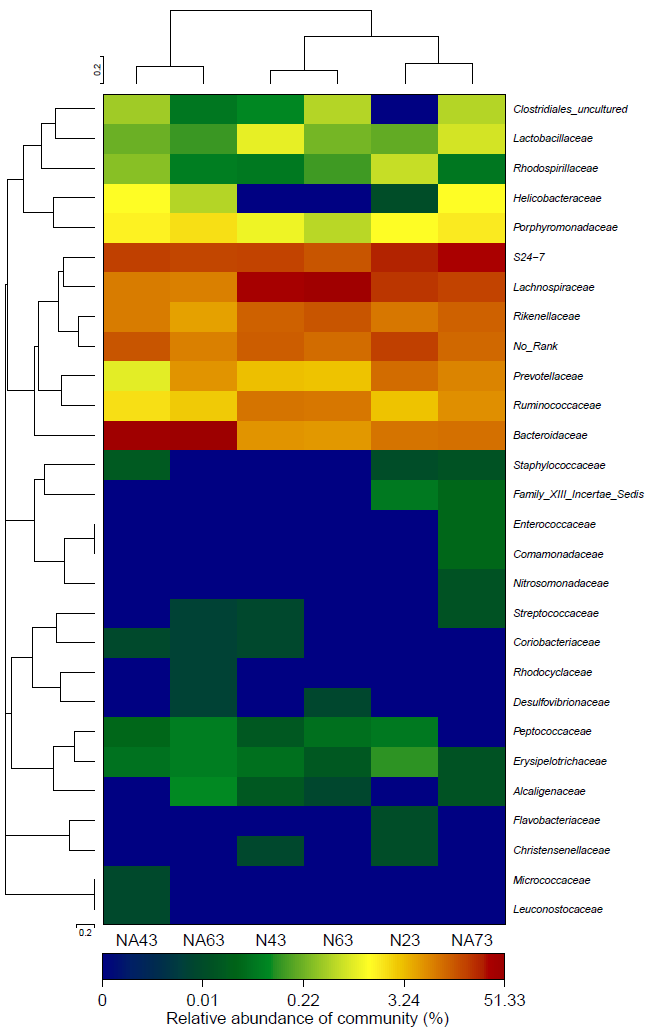


Figure B Comparison of colon microbial communities at family level
